# Iran Quality of Care in Medicine Program (IQCAMP): Design and Outcomes

**DOI:** 10.34172/aim.2023.21

**Published:** 2023-03-01

**Authors:** Saeid Shahraz, Sarvenaz Shahin, Yosef Farzi, Mitra Modirian, Nazila Shahbal, Mehrdad Azmin, Farnam Mohebi, Mohammadreza Naderian, Masoume Amin-Esmaeili, Naser Ahmadi, Shahedeh Seyfi, Hossein Zokaei, Roya Samadi, Bahram Mohajer, Roya Sherafat-Kazemzadeh, Abbas Balouchi, Bita Mesgarpour, Mahboubeh Parsaeian, Fatemeh Gorgani, Saral Rahimi, Sahar Saeedi Moghadam, Maryam Khezrian, Ahmad Amin, Shahab Baheshmat, Mohammad Reza Beyranvand, Majid Haghjoo, Mitra Mahdavi-Mazdeh, Masoud Mehrpour, Ghobad Moradi, Soheil Peiman, Besharat Rahimi, Afarin Rahimi-Movaghar, Reza Rikhtegar, Shahin Roshani, Mohammad Saadatnia, Seyed Mehdi Samimi Ardestani, Shahab Khatibzadeh

**Affiliations:** ^1^Tufts Medical Center, Institute for Clinical Research and Health Policy Studies, Boston, Massachusetts, USA; ^2^Non-Communicable Diseases Research Center, Endocrinology and Metabolism Population Sciences Institute, Tehran University of Medical Sciences, Tehran, Iran; ^3^School of Agriculture and Food Science, The University of Queensland, Brisbane, Australia; ^4^Haas School of Business, University of California, Berkeley, CA, USA; ^5^Tehran Heart Center, Cardiovascular Diseases Research Institute, Tehran University of Medical Sciences, Tehran, Iran; ^6^Department of Mental Health, Bloomberg School of Public Health, Johns Hopkins University, Baltimore, Maryland, USA; ^7^Psychiatry and Behavioral Sciences Research Center, Department of Psychiatry, Faculty of Medicine, Mashhad University of Medical Sciences, Mashhad, Iran; ^8^Institute for Global Health and Development, The Heller School for Social Policy and Management, Brandeis University, Waltham, Massachusetts, USA; ^9^Nursing Department, Iranshahr University of Medical Sciences, Iranshahr, Iran; ^10^Department of Pharmacology, School of Medicine, Tehran University of Medical Sciences, Tehran, Iran; ^11^Department of Epidemiology and Biostatistics, School of Public Health, Imperial College London, London, UK; ^12^Cardiogenetics Research Center, Rajaie Cardiovascular Medical and Research Center, Iran University of Medical Sciences, Tehran, Iran; ^13^Department of Neuroscience and Addiction Studies, School of Advanced Technologies in Medicine (SATiM), Tehran University of Medical Sciences, Tehran, Iran; ^14^Department of Cardiology, Taleghani Hospital, Shahid Beheshti University of Medical Sciences, Tehran, Iran; ^15^Cardiac Electrophysiology Research Center, Rajaie Cardiovascular Medical and Research Center, Iran University of Medical Sciences, Tehran, Iran; ^16^Iranian Tissue Bank and Research Center, Tehran University of Medical Sciences, Tehran, Iran; ^17^Imam Hossein Hospital, Shahid Beheshti University of Medical Sciences, Tehran, Iran; ^18^Social Determinants of Health Research Center, Research Institute for Health Development, Kurdistan University of Medical Sciences, Sanandaj, Iran; ^19^Department of Internal Medicine, AdventHealth Orlando Hospital, Orlando, Florida, USA; ^20^Advanced Thoracic Research Centre, Tehran University of Medical Science, Tehran, Iran; ^21^Iranian National Center for Addiction Studies (INCAS), Tehran University of Medical Sciences, Tehran, Iran; ^22^Institute for Diagnostic and Interventional Radiology, Alfried Krupp Hospital Ruttenscheid, Essen, Germany; ^23^Isfahan Neurosciences Research Centre, Alzahra Research Institute, Department of Neurology, Isfahan University of Medical Sciences, Isfahan, Iran; ^24^Departments of Psychiatry, Behavioral Sciences Research Center, Imam Hossein Hospital, Shahid Beheshti University of Medical Sciences, Tehran, Iran; ^25^Heller School of Social Policy and Management, Brandeis University, Waltham, Massachusetts, USA

**Keywords:** Cost of illness, Health care utilization, Iran, Non-communicable diseases, Protocol, Quality of health care

## Abstract

**Background:**

Assessment of quality and cost of medical care has become a core health policy concern. We conducted a nationwide survey to assess these measures in Iran as a developing country. To present the protocol for the Iran Quality of Care in Medicine Program (IQCAMP) study, which estimates the quality, cost, and utilization of health services for seven diseases in Iran.

**Methods:**

We selected eight provinces for this nationally representative short longitudinal survey. Interviewers from each province were trained comprehensively. The standard definition of seven high-burden conditions (acute myocardial infarction [MI], heart failure [HF], diabetes mellitus [DM], stroke, chronic obstructive pulmonary (COPD) disease, major depression, and end-stage renal disease [ESRD]) helped customize a protocol for disease identification. With a 3-month follow-up window, the participants answered pre-specified questions four times. The expert panels developed a questionnaire in four modules (demographics, health status, utilization, cost, and quality). The expert panel chose an inclusive set of quality indicators from the current literature for each condition. The design team specified the necessary elements in the survey to calculate the cost of care for each condition. The utilization assessment included various services, including hospital admissions, outpatient visits, and medication.

**Results:**

Totally, 156 specialists and 78 trained nurses assisted with patient identification, recruitment, and interviewing. A total of 1666 patients participated in the study, and 1291 patients completed all four visits.

**Conclusion:**

The IQCAMP study was the first healthcare utilization, cost, and quality survey in Iran with a longitudinal data collection to represent the pattern, quantity, and quality of medical care provided for high-burden conditions.

## Introduction

 The re-emerging concept of “value-based healthcare” has enabled the health systems to revise all aspects of healthcare in recent years.^1^ Health value is the quantity of the population’s health status improvement at the cost of promoting healthcare quality. The conceptual meaning of the value is to attain a certain amount of “quality” per change in the healthcare “cost”.^2^ Healthcare reformists have been increasingly noting the quantification of the quality and cost of health care services for some specific conditions.^1^ Knowing how patients with a particular disorder utilize the healthcare services throughout their healthcare journey is necessary to measure the quality of service. This needs data gathering systems and data infrastructure. Typical healthcare utilization studies in Iran and other developing countries often lack these essential data collection and reporting elements to capture the cost and quality accruing over a specific period.^3,4^

 While the nationwide assessment of the quality and cost of medical care has become a core health policy concern in developed countries, health authorities in developing nations have not entirely conceived of this concept and its application.^5^ Lack of consistent and reliable data on the quality and cost of services in developing countries has contributed to failed decisions to reform the healthcare systems.

 Recently, the Ministry of Health and Medical Education of Iran (MOHME) has emphasized measuring and monitoring the value of healthcare services at the population level, a subject that is above and beyond just the mainstream health services research.^6^ Therefore, we developed the Iran Quality of Care in Medicine Program (IQCAMP) to gather first-hand nationwide information on the quality and cost of care for a group of “medallion” medical conditions. Medallion conditions are the most prevalent and costly diseases with a significant burden worldwide. These conditions are acute myocardial infarction (MI), ischemic stroke, heart failure (HF), diabetes mellitus (DM), chronic obstructive pulmonary disease (COPD), major depressive disorder (MDD), and end-stage renal disease (ESRD).^7,8^

 Here, we present the study design and outcomes in detail to help replicate the study at scale. Methods and results describe the design and outcomes, respectively.

## Materials and Methods

###  Ethical Considerations

 We received Institutional Review Board approval for conducting the study from the “Deputy of Health” of MOHME. All patients were informed about the study steps, and verbal and written informed consent were obtained. The patients’ privacy precautions were fully considered. The next section conveys the details of our standards to respect the confidentiality of the data.

###  Conceptual and Analytical Framework

 This project comprises seven demonstration studies under the IQCAMP study. We borrowed the formulation of measuring the quality and cost of the services from the episode of care (EOC) concept. The EOC is a defined period to assess patients’ utilization patterns for a given condition.^9^ The EOC measures a particular problem from the patient’s first contact with the healthcare provider through the last encounter.^10-12^ We made estimations for three indicators: frequency, cost, and quality of given healthcare service for these seven conditions with this approach.

###  Target Population

 IQCAMP is a nationally representative cohort study. The measures of interest include patients’ utilization, cost, and quality profile with each of the seven conditions. The observation time equaled the EOC (any time after the anchoring time up to the end of the EOC).

###  Sampling Strategy

 We used a modified clustering sampling method. The details are as follows:

####  A. Province Selection

 The sampling method for selecting our representative provinces has been described in detail.^13^ In brief, being aware of the sample’s national representativeness and the high cost of sampling from all provinces, we planned to use a two-stage sampling design. In the first stage, we showed how a set of (health/healthcare) outcomes, deemed correlated with healthcare quality, vary across the provinces. The candidate outcomes are all-cause age-adjusted mortality rate, cause-specific mortality rate, inpatient and outpatient utilization frequency, achieved hemoglobin A1C level, body mass index (BMI), and systolic/diastolic blood pressure in patients with hypertension. We used data from death registration system in 2010 for the all-cause mortality rate and data from the 2005 Iranian Non-Communicable Disease Surveillance Survey (NCDSS) for the rest of the outcome measures. Our optimization model helped us select a group of provinces for sampling. The average of the estimated outcomes for the chosen provinces remained close to those of the national-level estimates.

**Figure 1:**
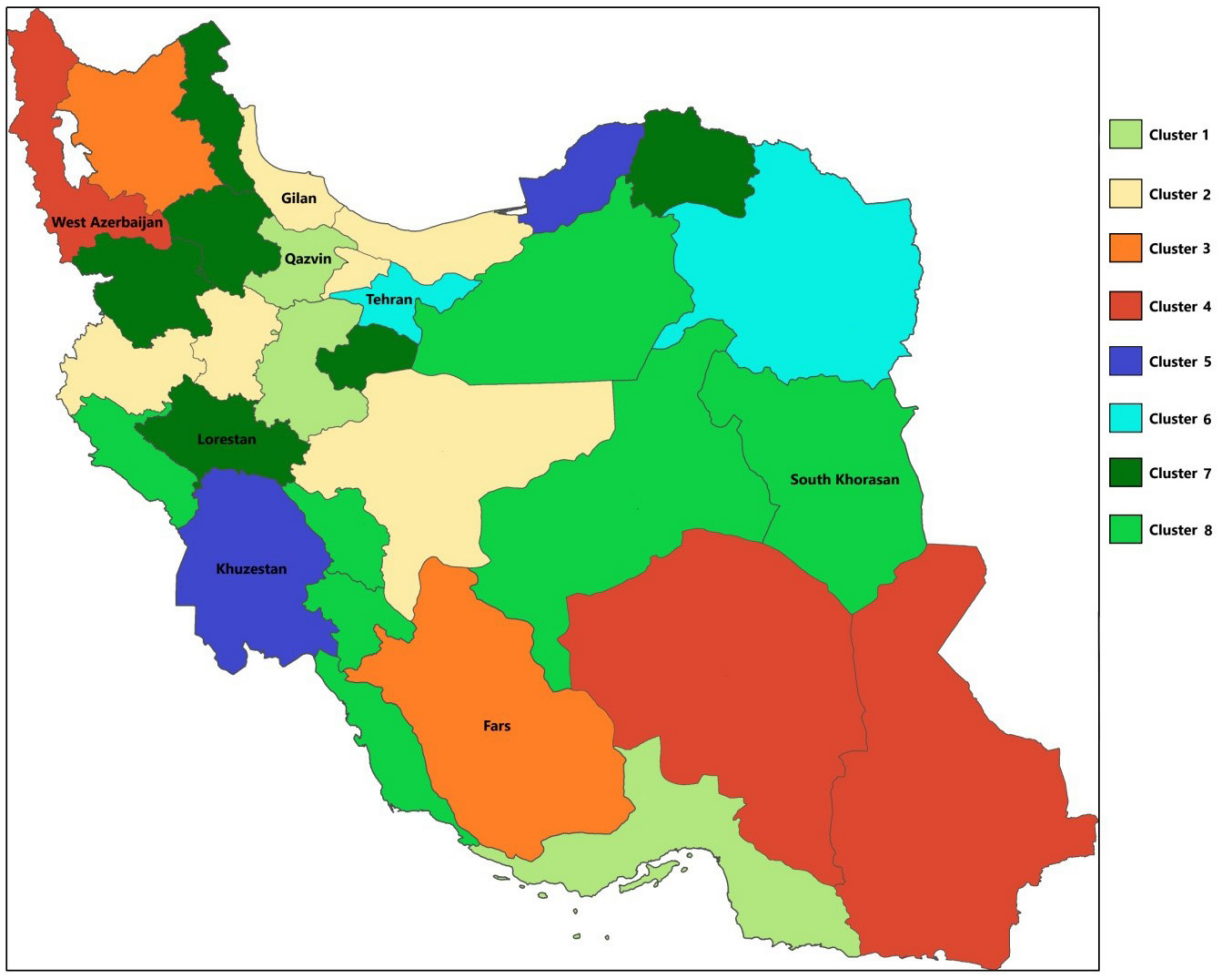
Map of the Eight Clusters in the Process of Province Selection and the Names of Selected Provinces

 We used a clustering data mining algorithm,^13,14^ which input the sub-province level information (outcomes) and treated each of the 31 provinces as a cluster. The algorithm identified provinces with similar estimated effects. Subsequently, the algorithm made up larger clusters by merging similar clusters. The super-clusters had the highest dissimilarity. The minimization algorithm outputs the best clusters representing the final national-level estimates. In the next step, the algorithm selected one of the provinces within each super-cluster (Figure 1). The selected province had the least distance from the center of the respected super-cluster. To validate the method, we randomly divided the sample into two subsets of training and test datasets. We tested the model based on the training dataset and then validated the model on the rest of the data (test dataset). The results of the two tests were similar.

####  B. Patient Selection

 Sample size calculation was challenging because of the unknown effect sizes for the various outcomes. The study budget was the principal limiting factor for extensive sampling. We planned to obtain hundreds of variables over several months from the same cohort of patients using self-reports on service utilization and abstract clinical information from patient charts and records, including outpatient, lab/imaging, and pharmaceutical. Gathering such detailed information limited us to sampling the smallest number of patients that only represent the entire nation. Because we targeted the samples from health services for six out of seven conditions (DM is the exception), we did not require large population samples for case findings. For diabetes, we used Iran’s 2016 STEPwise approach to NCD risk factor Surveillance (STEPS) population-based samples.^15^ We aimed to gather information from 300 patients per condition. In each target province, we chose the patients from one or two hospitals with a high patient referral rate.

###  Definitions and Case-Finding Method

 We used the standard definitions of the conditions as the starting point and developed working definitions to identify cases (Table 1). The formal definitions include a broader spectrum of the disease, where we focused on a narrower meaning to avoid misclassification. It was especially the case for chronic heart failure (CHF) and ESRD. We also had to compromise when we did not have enough equipment to test for required labs, such as the lack of enough spirometers for diagnosing COPD.

**Table 1:** Scientific and Working Definitions of the Medallion Diseases

**Disease**	**Scientific Definition**	**Working Definition**
Acute MI	Patients in which there is evidence of myocardial infarction along with elevation in ST segment in electrocardiogram, or an equivalent condition (such as new left bundle branch block), confirmed by abnormal cardiac biomarkers.^16^	Patients who their STEMI diagnosis was confirmed based on the scientific definition and clinical assessment of a cardiologist and were admitted to the CCU after the urgent treatment for STEMI (either receiving a fibrinolytic agent or undergoing primary percutaneous intervention).
HF	A complex clinical syndrome that results from any structural or functional impairment of ventricular filling or ejection of blood. This diagnosis includes various subgroups.^17,18^	Patients with clinical sign and symptoms of HF along with echocardiographic evidence of reduced ejection fraction (left ventricular ejection fraction less than 40 percent)- Diagnosed by a specialist according to the scientific clinical definition.
Ischemic stroke	An episode of neurological dysfunction caused by focal CNS infarction.^19^	Rapidly developed clinical signs of focal (or global) disturbance of cerebral function, lasting more than 24 h or leading to death, with no apparent cause other than of vascular origin, confirmed by diagnostic imaging techniques- Diagnosed by a specialist. Patients who were admitted due to the stated event for the first time, were included.
DM	Presence of FPG > 7 mmol/L or A1C > 6.5% (in adults) or 2hPG in a 75 g OGTT > 11.1 mmol/L or random PG > 11.1 mmol/L.^15,20^	Presence of FPG > 7 mmol/L or A1C > 6.5% (in adults), or past medical history of confirmed diabetes which is under treatment.
COPD	Patients with typical signs and symptoms of the disease with "post- bronchodilator ratio of FEV1 over forced vital capacity < 0.70".^21-23^	Patients with typical signs and symptoms of the disease, based on a specialist's clinical diagnosis, who are admitted to the hospital for controlling their symptoms.
MDD	MDD criteria from DSM-5.^24,25^	MDD patients according to DSM-5 criteria who were in the acute phase and whose initial treatment plan had been started or changed up to three months prior to the baseline interviews- Diagnosed by a specialist.
ESRD	The final stage of CKD, patients who have GFR of less than 15 mL/min and consequently need renal replacement therapy to survive.^26,27^	Patients who undergo hemodialysis.

MI, myocardial infarction; HF, heart failure; DM, diabetes mellitus; COPD, chronic obstructive pulmonary disease; MDD, major depressive disorder; ESRD, end-stage renal disease; CNS, central nervous system; FPG, fasting plasma glucose; OGTT, oral glucose tolerance test; 2hPG, 2-hour plasma glucose; PG, FEV1. plasma glucose; forced expiratory volume in 1 s; CKD, chronic kidney disease; CCU, coronary care units; DSM-5, Diagnostic and Statistical Manual of Mental Disorders; GFR, glomerular filtration rate.

###  Defining the Measures and Sources of the Data

####  1. Utilization

 We assessed the utilization of health services for each condition separately. The utilization assessment included various services, such as hospital admission, outpatient visits, diagnostic and laboratory tests, rehabilitation, medicines, medical equipment, and home care services. Patients reported the quantity of service utilization at each interview.

####  2. Cost of Care

 The method for estimating direct medical costs is explained further in detail. Direct non-medical costs consisted of the costs for transportation, food and hoteling, and childcare. Indirect costs were the ones that caused lower earnings for the patients. We used three methods to estimate self-reported indirect costs: time wasted in waiting rooms, absenteeism from work, and productivity loss (lower productivity of the patients at work due to their disease). The days that the patients (or the family members) reported being absent from work were multiplied by the minimum daily wage according to the work law in Iran. We acknowledged that the cost of illness in Iran, regardless of estimation methods used, would be underestimated due to a lack of archived documentation on an itemized substantial governmental subsidy and donations to [public] healthcare providers. Estimating governmental and public share to build, equip, and maintain hospitals or clinics is challenging due to poor documentation of these costs. For example, kidney dialysis centers receive monetary assistance through government and advocacy groups on an ad-hoc basis.

 Direct medical costs had two sources: patients’ self-reports and invoices (bills). The categories of expenses included inpatient services, diagnostics, laboratory, outpatient visits, home care, equipment, medication, and rehabilitation. Out-of-pocket (OOP), insurance-paid, total, unofficial, and other costs were separately recorded for each group. We employed two approaches to analyze the sensitivity of this method of recording (self-report and invoice). First, we listed all the services used for each patient and calculated the total cost based on the national tariffs for each service unit to compare with our records. The other method was to compare the costs collected from self-report and invoice to evaluate their agreement. We used the intraclass correlation coefficient (ICC) and found good consistency (ICC > 0.7) in both techniques for total costs, thus confirming the desired sensitivity of this method for documenting costs.

**Figure 2:**
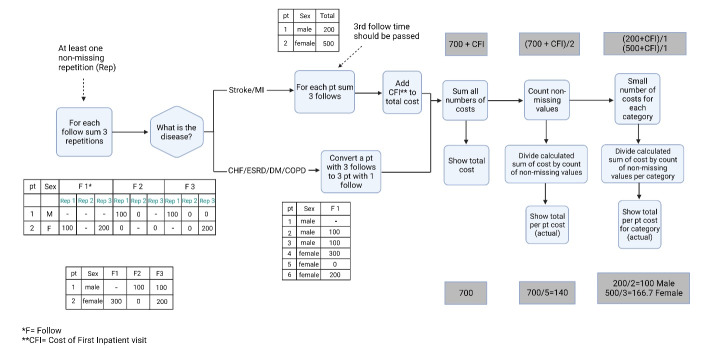
Cost Calculation Algorithm – Explanation: Each patient had three follow-up visits. The interviewer asked patients to report their last month’s medical costs. The patients had the option to report the costs for up to three encounters. For MI and stroke, whose episodes began with an inpatient, we calculated the seasonal cost as the monthly cost of the disease after the episodes would vary with the intensity of services they received. The cost per episode of MI or stroke was the sum of the hospitalization cost and cost of care for three consecutive follow-up months. For all other diseases, we assumed slight month-to-month variation and scaled up the total cost of three months of care to get the annualized cost

 We considered four interviews in the study protocol, one baseline interview, and three follow-up interviews. During each follow-up, we recorded the reported costs. For all diseases except MI and stroke, whose episode was initiated with hospitalization, we assumed a steady utilization and cost during a year. Therefore, we calculated the average monthly costs for each disease (Figure 2). For MI and stroke, we added the initial cost of hospitalization to the follow-up costs and reported a 3-month cost for the entire acute episode (Figure 2).

####  3. Quality of Care

 We broadly reviewed the available quality measures to assess the quality of care for each condition. Our first source of measure review was the measures set forth by the National Quality Forum (NQF). We also scanned and selected the quality indicators from other credible organizations. For instance, the Agency for Healthcare Research and Quality (AHRQ) holds a collection of measures in the National Quality Measures Clearinghouse (NQMC).^28^ The eligibility criteria for the indicators were A) Scientific acceptability, B) Reliability and reproducibility, C) Relevance, and D) Feasibility. Quality indicators were carefully selected for each condition by a committee of clinician experts in the respected category. Due to the divergent nature of the services provided for each disease, we designated multiple indicators per each condition. Table S1 (Supplementary File 1) shows the final set of measures chosen by our experts.

####  4. Medication Use and Adherence

 Assessment of medication adherence requires a standard instrument. Lavsa et al have systematically reviewed the literature to provide an updated list of the tools for measuring medication adherence.^29^ Pill count and patient self-reports^30^ were our methods of choice for adherence measures. Physicians in Iran enter the prescription orders in patients’ insurance notebooks. Each pharmacy encounter includes three identical copies of the same prescription. We prepared two forms for medication data collection. The first form helped collect information written on the first page of the prescription notes. This form was specific for each of the following conditions: CHF, COPD, AMI, and stroke. We customized the second form for non-episodic conditions, i.e., DM, MDD, and ESRD. Both forms required generic drug names, medication dosage, the units prescribed, and the route of administration.

 The drugs were counted during each appointment to assess their adherence to the prescription. We asked the patients to bring all their medication boxes during the visits to avoid misunderstandings and mistakes regarding the medicines. We gave them a pill box and asked them to get all their prescriptions and drugs for each follow-up. The interviewers filled in the medication questionnaires based on the counted medications, prescriptions, and other related documents. Medication questionnaires included information on the generic names of the medicine, the form of medicine, dosages, units, and intake frequency.

###  Project Management 

 We employed a participatory approach by developing multiple teams for project management. Table 2 conveys the details of each working group.

**Table 2:** Project Management: The Breakdown of Executive Teams and Their Responsibilities

	**Members**	**No. of Members**	**Responsibilities**
Core team	Medical specialists Pharmacists Epidemiologists Operational manager Field managers Statisticians	11	Drafting the main policies of the project and decision making Organizing the project Controlling the outputs
Expert committee	Medical specialists, epidemiologists, and statisticians, all with research history in each medallion condition	23	Reviewing international guidelines and quality measures Comparing international guidelines with the standards currently implemented in Iran. Defining the elements of each EOC Designing specialized questionnaires
Coordinators	Medical doctors	40	Coordinating among the specialists within each committee Coordinating the core team and expert committees when designing the questionnaires Monitoring the performance of provincial interviewers
Provincial specialists	Medical specialists	156	Suggesting target hospitals Patient selection
Data collection team	Nurses	78	Interviewing the participants based on the questionnaires
Data analysis	Statisticians	3	Data cleaning Data analysis

EOC, episode of care.

1. The core team was responsible for drafting the central policies of the project, organizing it, and controlling the outputs.
2. Expert committees: The core team formed specialized groups for each disease and an additional group for cost analysis. We chose specialists with outstanding research backgrounds specific to each condition. Hence, we developed seven condition-focused scientific committees. The expert committees were responsible for reviewing international guidelines and quality measures to compare them with the standards currently published for the clinical sector in the country. They defined the elements of the episode and designed a specialized questionnaire to collect patient information about the utilization, quality, and cost of care. Each committee had discretionary authority for modifying methods but not the study aims.
3. The coordinators oversaw the project operation with little protocol deviation. The core team selected one coordinator for each expert committee to make the necessary coordination between the committee members and the core team for designing the questionnaires. The coordinators were also responsible for monitoring the interviewers’ performance and answering their questions.
4. The provincial medical specialists helped recruit patients from medical centers at the province level.
5. The data collection team comprised trained nursing staff selected from each designated medical center to conduct the interviews. The nursing staff was already in direct contact with the recruited patients.
6. The data analysis team functioned under the guidance of the core team; this working group comprised statisticians, data scientists, and data engineers. The team was responsible for developing the software, programming, data cleaning, and analysis.

###  Developing Questionnaires

 We designed and developed multiple modules of a questionnaire to gather demographic and baseline information at the start of the study, service utilization within the specific period, quality of health care services received, and cost per service during the episode. We also developed and received expert endorsement for a tool to collect relevant data to measure medication adherence in Iran.

 The core and expert teams jointly devised the draft version of the questionnaires using the existing guidelines. Next, the experts evaluated the content and face validity of the questionnaires, addressed the critical issues, and updated the draft to a semi-final version. A ten-person cognitive debriefing interview revealed the questions’ understanding and clarity problems and helped draft the final version of the questionnaires. The final versions comprised four modules:

1. Demographic information: This module was the same for all the diseases.
2. Baseline information: These modules were specifically designed for each disease and included questions about the patients’ awareness of their disease, their current disease status, complications, and previous health services received regarding their disease (services used by the patients before the beginning of the study), and the factors that had affected their course of treatment. In addition, the validated Farsi version of EuroQol-5d^31^ was used to assess the patients’ general health status and quality of life.^32^
3. Follow-up for utilization of healthcare services and the quality of care: The disease-specific modules included questions about the details of services utilized during the last month(s), both in outpatient and inpatient settings. The questions were developed to assess the utilization and quality of care, completed by patients’ self-reports and using their medical files and documents. The outpatient services included doctor visits, laboratory tests, diagnostic imaging, medications, and rehabilitation and supporting services. (For more information, see “Defining the measures and sources of the data.”)
4. The medications were recorded in the medication adherence questionnaires as part of health services modules. This sub-module was for the prescription records and was available through insurance claims.
5. Cost of healthcare: Estimating the cost of services was one of the critical objectives of this survey. Healthcare cost modules were developed to gather data on the direct medical cost and the share of insurance companies and patients (out-of-pocket payments). In addition, data on direct costs (medical and non-medical) of disease and indirect costs were collected separately. The costs of inpatient and outpatient services were recorded, detailed in the date and location of the services. (For more information, see “Defining the measures and sources of the data.”)

###  Data Collection

####  Information Technology

 We used computer-assisted patient interviews throughout the study, including a period after the completion of the last interview for missing information data recollection. We developed an Android application using Java programing language and MS-SQL for database maintenance. Data gathering involved two main steps: 1- a RESTful web service designed to manage data, and 2- a process-based Android application developed for data gathering. Our application had the capability of both offline and online data gathering. Our interviewers could collect data offline; data would be stored on the local application database (SQLite) and sent to the server when the device could find an internet connection. A dashboard was developed for the core team to manage study users, patients, processes, and reporting.

 We used a secure path for communication between users and the database. We applied RSA (Rivest-Shamir-Adleman) Encryption method using HTTPS and established an SSL (Secure Sockets Layer) protocol for a secure channel. We put our database server inside DMZ (De-Militarized Zone) to build an efficient system with appropriate access, with two layers of WAS (Websphere Application Server) and DB (Database). This architecture could store data on the database at a standard security threshold.

####  Training Interviewers

 To select and train the interviewers, we adopted a stepwise approach. First, the coordinators of each province provided a list of nurses from target hospitals and clinics. The final number and list of nurses were determined based on the sample size of each disease group, the number of selected health service centers, average interview duration, the number of patients a nurse could interview within a week, the nurse’s experience related to the specified condition, and approval of their department head for their research experience and clinical seniority.

 The training logistics were as follows: 1. Developing educational material about the study, including the specified conditions, the questionnaires, the interview guide, and the follow-up processes; 2. Information technologies needed to conduct the training sessions; 3. Provision and purchase of the required equipment to be delivered to the nurses during the training courses, including medication-holding bags and document folders, the tools needed for counting the patients’ medicines, tablets, and smartphones required for communication and recording the data; and 4. Travel arrangements for the teams to participate in the workshops.

 Subsequently, we set up a pilot workshop in South Khorasan to evaluate and receive the interviewers’ feedback on the training sessions and logistics. We carried out six one-day workshops at the Non-Communicable Diseases Research Center of Iran (NCDRC) in Tehran to train all nurse interviewers. The interviewers were divided into seven condition-specific groups and received separate training tailored for each of the seven conditions. During the training workshops, the interviewers received training on the study protocol, survey objectives and process, questionnaires, and interview instruction. They also received detailed instructions on data collection and storage software. Each trainee ran usability testing on a few patients to complete the training and troubleshooting.

####  Interviewing Process

 To this end, we developed mobile/tablet-friendly applications to facilitate real-time secured data collection and storage. We employed electronic questionnaire technology for data collection. The data team provided 24/7 interviewer support to ensure a seamless data collection process. We received the list of potential study participants from the specialists at each study site who agreed and signed consent to provide patients’ contact securely.

 The index event for acute MI and stroke was first-time hospitalization. The participants received detailed instruction on the study objectives and their right to leave the study at will and during the study; the consenting participants were recruited. The interviewers contacted each patient from the list for HF, ESRD, COPD, and MDD. The interviewers received instruction to call patients three times to increase the response rate. For DM patients, the entire data collection was conducted by phone.

 During the first visit, the interviewers thoroughly explained the project to the patients and taught them the study regulations. Furthermore, the interviewers collected demographic information, the disease’s current status, and history.

 We conducted three follow-up interviews at one-month intervals for all the conditions except MDD. For the latter, a two-month follow-up interview was planned. Patients provided hospitalization records for the verification of self-report in-patient events. The participants agreed to bring their medication boxes in subsequent visits for medication count.

###  Analysis Plan 

 In order to reduce missing data points, all patients were contacted again (via telephone), and their names, national ID codes, ages, and education levels were rechecked. We devised data cleaning steps as part of the statistical analysis plan, emphasizing the cost of data outliers. In doing so, we calculated the mean of cost by excluding one observation at a time and omitting values that generated a mean beyond the 95% confidence interval of the mean. See below for a relatively detailed description of cost estimations. Patients were the unit of analysis for all modules except for the quality indicators. For these indicators, the quality was the fraction of patients who received a service for which they were eligible. The statistical methods were limited to central tendency and dispersion metrics, including mean, standard deviation, and ratios. Principal component analysis was the method to calculate income deciles using population assets. We used a two-way absolute agreement ICC metric^33^ to estimate the measurement equivalence between costs reported by self-report and invoice. Upon achieving enough agreement (lower band of 95% confidence interval of the ICC > 0.7,^34^ we could interchangeably use the cost estimates by self-report or invoice to reduce missing values. To avoid identifying healthcare providers, we did not plan to report any sub-national results.

####  Cost Calculations 

 Inpatient, diagnostic, laboratory, outpatient, home services, equipment, medicine, and rehabilitation costs contributed to medical expenses estimates. We collected data on the share of out-of-pocket payments, insurance coverage, and total expenses separately. We also asked patients to provide information on informal payments they made during the specified EOC. A printed cost diary format was provided to the patients at the first interview and each follow-up. Furthermore, another form was provided to be used alongside insurance notebooks of patients while visiting pharmacies since most pharmacies only include the total payment and not the granularity needed in this study, i.e., OOP, insurance, and total cost.

 The OOP, Insurance, and total costs were gathered using two methods. The first one was accessing the invoice issued by the service provider whenever available. These invoices included all the needed information for IQCAMP. Self-reported costs were also gathered for services with and without invoices as the second method. These methods helped develop an inclusive cost profile for each patient. We added up the costs of services received by each patient per category and then added up all categories to calculate the total cost per patient. ICC analysis found acceptable consistency (ICC > 0.7) between the two methods in estimating total costs.

 For direct non-medical expenses, patients reported the cost of transportation, food, accommodation, and childcare for the duration of the illness. Indirect costs were estimated for wasted waiting time and lost or reduced income. At the time of follow-up visits, the interviewers asked patients to answer the cost module questions to ensure the cost reports’ continuity. Figure 2 shows the practical stepwise approach taken for cost calculations.

 We calculated the seasonal costs for diseases that start with an acute episode (i.e., MI and stroke) and had initial hospitalization. We extrapolated the annual costs for chronic diseases (i.e., CHF, ESRD, DM, MDD, and COPD).

###  Quantifying Medication Use

 We converted all medication names to their generic names. Patient Daily Dose (PDD) was calculated as the number of a specific medicine taken at each dose x daily intake frequency x unit (dosage). For instance, PDD for a patient receiving one 500 mg metformin pill three times a day would be 1500 mg. We calculated and recorded PDD for all medications a patient received. Then, for each group of diseases, the final descriptive table of PDD provided summary statistics, including the number of cases, PDD means, median, standard deviation, and range. Cases with missing data on medication dosage or forms were excluded.

## Results

 The IQCAMP study was conducted as a collaboration between eight medical sciences universities and 27 selected hospitals. The provinces were Qazvin, Gilan, Fars, West Azerbaijan, Khuzestan, Tehran, Lorestan, and South Khorasan. Seventy-eight trained nurses conducted the interviews and entered the data, and 156 physician specialists (in all seven disease subgroups) helped with patient identification and recruitment. Table 3 details the number of nurses in each cluster and disease subgroups.

**Table 3:** Number of Nurses (Patients) Per Clusters and Diseases

**Cluster/Diseas**e	**AMI**	**HF**	**Stroke**	**DM**	**COPD**	**MDD**	**ESRD**	**Total**
Qazvin	1 (15)	0 (0)	1 (18)	2 (7)	1 (1)	1 (17)	1 (18)	6 (76)
Gilan	1 (65)	2 (60)	1 (66)	3 (23)	3 (50)	2 (65)	2 (65)	13 (394)
Fars	1 (33)	1 (18)	2 (34)	1 (30)	1(34)	1 (32)	1 (34)	8 (215)
West Azerbaijan	1 (35)	1 (35)	1 (35)	2 (25)	1 (21)	1 (35)	1 (35)	8 (221)
Khuzestan	0 (0)	1 (7)	1 (15)	2 (22)	1 (25)	1 (23)	1 (24)	6 (116)
Tehran	3 (64)	7 (57)	3 (34)	4(40)	3 (64)	5 (41)	4 (83)	22 (383)
Lorestan	1 (12)	1 (9)	2 (9)	2 (4)	1 (8)	1 (29)	1 (30)	8 (101)
South Khorasan	2 (24)	2 (23)	1 (23)	1(6)	2 (32)	1 (23)	1 (24)	7 (155)
Total*	10 (248)	15 (209)	12 (234)	17(157)	13 (235)	13 (265)	12 (313)	78 (1661)

MI, myocardial infarction; HF, heart failure; DM, diabetes mellitus; COPD, chronic obstructive pulmonary disease; MDD, major depressive disorder; ESRD, end-stage renal disease. * Some nurses covered more than one disease in each province.

 The IQCAM core team held more than 300 meeting hours to manage the study and develop and conduct the pilot study. The team spent nearly 120 hours training nurses during one or two-day workshops. While the target sample size for each condition was 300 patients to achieve enough power for all objectives, we could gather only a partial sample for most diseases. Diabetes patients constituted the lowest (N = 157), and ESRD patients made up the highest sample size (N = 313). The recruitment was the highest in South Khorasan (99% of the target sample size) and the lowest in Lorestan (slightly over50% of the target sample size).

###  Demographic and Patient Characteristics

 A total of 1661 patients initially participated in the study, and 1097 patients completed all four visits. Of 209 HF patients, 60.3% were males. The age groups 36–65 and > 65 years comprised 51.2% and 35.9% of the subjects, respectively. We followed 248 acute MI patients. Over two-thirds (174 patients) of them were between 36 and 65 years of age, and four out of five were males (79.1%). Moreover, 1.6% of our MI cases were younger than 35 years. Among 234 (54.3% males) stroke patients, 124 (53.0%) were older than 65. Most of the COPD cases were males (65.5%), almost equally distributed between the two age groups of 36-65 and > 65. We recruited 313 (57.8% males) ESRD patients, most (54.3%) of whom were in the age group 36–65 years. A significant proportion of MDD patients were women (73.6%). These patients were generally younger than patients in other groups, and only 5.3% were older than 65. Patients with DM (N = 157, 42.7% males) were mostly in two age groups: 61.8% were between 36 and 65 years, and 28.7% were over 65. Table 4 includes more details on patient demographics. The team will publish disease-specific results on healthcare utilization, quality, and cost in separate publications.

**Table 4:** Demographic Characteristics of IQCAMP Participants

**Characteristics**		**Acute MI**	**HF**	**Ischemic stroke**	**DM**	**COPD**	**MDD**	**ESRD**
Total participants		248	209	234	157	235	265	313
No. of the participants who completed all four visits		187	146	153	90	114	164	243
Age, No. (%)	18–35	4 (1.61%)	8 (3.83%)	1 (0.43%)	3 (1.91%)	1 (0.43%)	75 (28.3%)	23 (7.35%)
	36–65	174 (70.16%)	107 (51.2%)	88 (37.61%)	97 (61.78%)	100 (42.55%)	168 (63.4%)	170 (54.31%)
	65 <	69 (27.82%)	75 (35.89%)	124 (52.99%)	45 (28.66%)	118 (50.21%)	14 (5.28%)	115 (36.74%)
Gender, No. (%)	Male	196 (79.03%)	126 (60.29%)	127 (54.27%)	67 (42.68%)	154 (65.53%)	70 (26.42%)	181 (57.83%)
	Female	52 (20.97%)	83 (39.71%)	107 (45.73%)	90 (57.32%)	81 (34.47%)	195 (73.58%)	132 (42.17%)
Literacy, No. (%)	Illiterate	45 (18.15%)	50 (23.92%)	70 (29.91%)	31 (19.75%)	57 (24.26%)	37 (13.96%)	42 (13.42%)
	Elementary school	58 (23.39%)	47 (22.49%)	56 (23.93%)	28 (17.83%)	47 (20.0%)	54 (20.38%)	54 (17.25%)
	Middle/High school	42 (16.94%)	24 (11.48%)	23 (9.83%)	21 (13.38%)	21 (8.94%)	61 (23.02%)	33 (10.54%)
	Diploma	31 (12.5%)	29 (13.88%)	22 (9.4%)	28 (17.83%)	26 (11.06%)	62 (23.4%)	59 (18.85%)
	Associate/Bachelor’s degree	20 (8.06%)	10 (4.78%)	7 (2.99%)	11 (7.01%)	5 (2.13%)	40 (15.09%)	35 (11.18%)
	Master’s/Doctoral degree	7 (2.82%)	2 (0.96%)	3 (1.28%)	2 (1.27%)	2 (0.85%)	7 (2.64%)	4 (1.28%)
	Post-graduate degree	0 (0%)	0 (0%)	0 (0%)	0 (0%)	0 (0%)	1 (0.38%)	0 (0%)
	Religious school	0 (0%)	0 (0%)	0 (0%)	0 (0%)	1 (0.43%)	0 (0%)	1.0 (0.32%)
Wealth index, No. (%)	Very low	63 (25.4%)	35 (16.75%)	93 (39.74%)	9 (5.73%)	56 (23.83%)	20 (7.55%)	57 (18.21%)
	Low	39 (15.73%)	39 (18.66%)	44 (18.8%)	46 (29.3%)	71 (30.21%)	37 (13.96%)	56 (17.89%)
	Intermediate	33 (13.31%)	51 (24.4%)	50 (21.37%)	39 (24.84%)	61 (25.96%)	46 (17.36%)	52 (16.61%)
	High	54 (21.77%)	45 (21.53%)	20 (8.55%)	41 (26.11%)	31 (13.19%)	80 (30.19%)	61 (19.49%)
	Very high	59 (23.79%)	39 (18.66%)	27 (11.54%)	22 (14.01%)	16 (6.81%)	82 (30.94%)	87 (27.8%)
Deceased		0	6	8	1	1	1	7

MI, myocardial infarction; HF, heart failure; DM, diabetes mellitus; COPD, chronic obstructive pulmonary disease; MDD, major depressive disorder; ESRD, end-stage renal disease.

## Discussion

**Figure 3:**
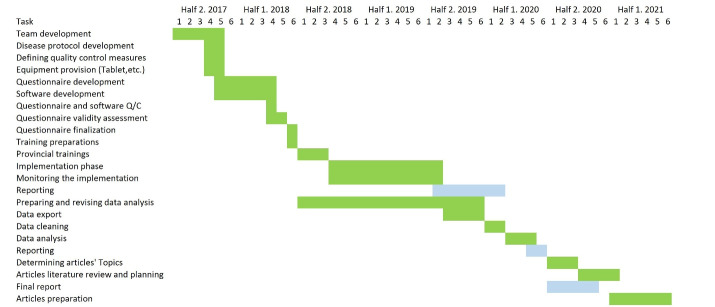
IQCAMP Project Timeline

 We completed the recruitment of patients for 1661 patients, equal to 79.1% of the initial target sample. Of these, 1097 (66.0%) completed the last follow-up. Our subjects were mainly from public hospitals affiliated with academic centers. However, we included two centers from the social security organization and one large private center in Tehran. Of note, private healthcare centers and those not affiliated with MOHME’s academic centers have strict data and patient policies. These policies caused a delay in recruitment. To our knowledge, IQCAMP is the first all-payer healthcare research in Iran on the national scale. Table 5 and Figure 3 show the timeline of different study steps.

**Table 5:** IQCAMP Project Timeline

**Steps**	**Time (mon)**
Design and development	
Developing the management and expert teams	2
Developing the other teams including, provincial, IT and analysis	3
Developing sampling method	2
Developing questionnaires	6
Preparing and logistics	
Recruiting nurse interviewers	1
Educating interviewers	3
Preparing the materials	2
Conducting the interviews	
Recruiting patients	2
Following up	9
Reporting	
Cleaning the data	2
Analyzing the data	3
Preparing the reports and articles	14

 We conducted this study without prior information about healthcare utilization, cost, and quality of services in Iran. These data sources are not available as part of a national effort to gather and clean healthcare administrative data at the national level. Hence, we consider this study an attempt at developing such information by applying a cost-efficient and systematic method. The longitudinal nature of data collection is a unique feature of IQCAMP. Previous studies have used a cross-sectional design with an extended recall time. This study collected information with a short recall period (one month) to minimize recall bias. Also, the panel nature of data collection enabled us to validate the cost claims by inspecting the administrative documents from healthcare providers or payers.

 The NCDRC provided IT support, interviewer training, data scientists, and a host of facilities to conduct the study. The study’s management team invited research experts and specialists to form a scientific steering group for each disease via a participatory approach. The disease expert groups participated in the research protocol and questionnaire design. We piloted the questionnaires on a small number of patients for their fluency, accuracy, and usability. During the sampling stage, expert groups and the software team provided uninterrupted scientific and technical support for the interviewers. Selecting nursing staff providing care for the patients as interviewers helped us develop trust between the interviewers and the patients. We trained the nurse practitioners via intensive one-day training in Tehran or the target cities.

 The senior project manager could communicate with the expert groups and interviewers via a coordinator assigned to each expert group and medical specialist. The coordinators were among medical residents or students with a research background and received essential training and guidance on operationalizing the study.

 We adopted all three views for cost estimation, i.e., patient, payer, and society. To provide necessary cost components, we imputed patient and payer shares. We collected various data on incurred direct non-treatment and indirect costs to complement the cost analysis.

 We did not plan to determine the government’s share of healthcare costs as there is little transparency and available documentation on how various sources of government funds as global payments for public providers in the country are released. The government releases financial assistance to most providers on an on-demand basis and when they struggle economically. These boosting monetary funds are not traceable. Examples are subsidies for medicine and medical equipment, assistance in expanding hospital facilities, and financial assistance to compensate for unpaid salaries of medical staff that are often delayed for months or more than a year at the time of financial hardship. Access to healthcare provider ledger books to single out governmental sources of financial support for healthcare providers is next to impossible. Also, medical bills shared with patients did not contain detailed information to support our cost analysis as a single source.

 Conducting a longitudinal national survey using a limited research fund is challenging. We could not report the detailed results by geography because of the small sample size and the risk of identifying the centers from which we found our patients. We showed that our interviewers achieved up to a 67% completion rate in gathering patient information within three follow-up periods. We predict a shorter turnaround time for the study results and a higher completion rate in future studies rendered by the government to establish sufficient logistical support. Overall, we fulfilled the study’s aims by devising an innovative sampling strategy using machine learning methods.^13^ A well-funded similar study at the national level may not require our sampling strategy if each province receives sufficient financial and administrative support to gather province-level data.

 We advise the reader to use caution in interpreting the result of our study:

1. The definition of conditions matches a specific spectrum of disease severity.
2. The study’s limited statistical power precludes subgroup comparisons.
3. Although partly verified by administrative sources, the self-report nature of the study brings about a few biases, such as recall decay.
4. The volatile nature of inflation because of longstanding economic hardship should be accounted for when interpreting the cost estimates.

In conclusion, the IQCAMP study, as a national demonstration study, aimed to open a new chapter of research in the field of health services in Iran. This study is a blueprint to guide collecting public data on the utilization, cost, and quality of health services, especially for high-priority medical conditions. This research typically enables researchers to provide better estimates of underused, inappropriately used, and overused medical services. It can also help formulate policies for improving the value of healthcare services. This study informs the design of a periodic (e.g., every five years) large-scale national and subnational study to monitor the cost and quality of services for high-cost high-burden diseases. As an alternative, the IQCAMP team posits that in the absence of enough resources to support the periodic surveys, this study can guide extending the quality and quantity of disease-specific registries. Adding IQCAMP modules to the existing national disease registries enriches the registry with a wealth of complementary data on quality and cost of care specific to the target therapeutic area.

## Supplementary File

Supplementary file 1 contains Table S1.
