# Supplementary material for: Iran Quality of Care in Medicine Program (IQCAMP): Design and Outcomes
Source: Arch Iran Med. 2023 Mar 1;26(3):126–37. doi: 10.34172/aim.2023.21 (PMC10685727; doi:10.34172/aim.2023.21)
Supplement: Supplementary file 1 — contains Table S1. [file aim-26-126-s001.pdf]

Arch Iran Med. March 2023;26(3):126-137

10.34172/aim.2023.21

http://journalaim.com

Supplementary file 1

Table S1. The quality-of-care indicator questions regarding each medallion disease

Disease	Quality indicator questions
**Acute myocardial infarction (Acute MI)**	• How long (minutes) did it take for the AMI patient from attending the ER to be admitted?<br>• If primary PCI* has been done for this patient, how long (minutes) did it take from entering the ER to inflation of the balloon in the occluded artery?<br>• If IV fibrinolytic drugs have been prescribed for the patient in the ER**, how long (minutes) did it take from attending the ER to inject the drug?<br>• If there was no contraindication, did the patient receive asprin (in the form of four 80 mg tablets, or three 100 mg or a 325 mg tablet)?
**Heart failure**	• Is echocardiography performed and the ejection fraction (EF) measured for this patient during admission?<br>• If EF was under 40, has the patient received Beta-blocker?<br>• If EF was under 40, has the patient received Angiotensin Receptor Blocker (ARBs) or Angiotensin Converting Enzyme Inhibitor (ACEIs)?
**Ischemic stroke**	• Were antithrombotic medications (A.S.A 325, Plavix [Clopidogrel], (or OR and?) Dipyridamole) prescribed for the patient in the first 24-48 hours?<br>• Was the patient discharged with an antithrombotic medication?<br>• Was the patient discharged with a Statin?<br>• Has the patient developed dysphagia (of solids or liquids) after the stroke?<br>• During the admission, did the patient receive any type of rehabilitation services?<br>• Did the patient receive Heparin, Enoxaparin, Apixaban, or Rivaroxaban during admission to prevent DVTs[†]?<br>• Has your neurologist (stroke doctor) talked to or asked about cigarette smoking?<br>• When the stroke happened, was the patient transferred to the nearest hospital by EMS[††]?<br>• Did the patient receive IV[‡] tPA[‡‡] treatment?
**Diabetes mellitus**	• During the last year, have you done a laboratory testing of your blood sugar?<br>• During the last year, has your doctor examined your feet to look for ulcers and injuries?<br>• Has your doctor explained appropriate footwear for a diabetic person?<br>• Has a doctor or healthcare staff talked to you about smoking cessation?

	• In your latest visit, did your doctor (or other healthcare staff) talk to you about the importance of controlling your blood sugar and blood pressure and the complications attributed to these measures?<br>• Has your doctor (or other healthcare staff) given you a booklet or a guide about your disease (diabetes) that you can refer to in case of having a question?<br>• During the last year, has a doctor (or other healthcare staff) talked to you about the appropriate diet for diabetes control?<br>• During your visits, have your doctor/nurse/behvarz§ talked to you about physical activity and appropriate workouts?<br>• In your latest visit, did your doctor (or dietician) measure your weight?<br>• Has your doctor taught you what to do in case of having hypoglycemia (dropping your blood sugar)?
**Chronic obstructive pulmonary disease**	• When you first received the inhaled drugs, did your doctor teach you how to use the inhaler, and did he/she check how you used it practically?<br>• During the last year, has your doctor assessed how you use your inhaler?<br>• After your latest exacerbation of symptoms, has your doctor assessed how you use your inhaler?<br>• During your latest hospital admission (due to this disease), did your doctor recommend you to do rehabilitation for four weeks?<br>• After your latest hospital admission (due to this disease), did you start rehabilitation?<br>• Did you complete your rehabilitation program for four weeks?<br>• In the patient's latest visit, was his/her O2 saturation between 88 to 92?<br>• Does the patient have spirometry results?
**Major depressive disorder**	• (Ask the doctor) Have you asked the patient about maniac and hypo-maniac episodes?<br>• (Ask the doctor) Have you asked the patient about a history of drug use, alcohol, and related disorders?<br>• Did your doctor ask you about your suicidal thought ?<br>• Was any medication prescribed for your depression?
**End-stage renal disease**	• **Was** the patient's hemoglobin above 11 g/dl during the past month?<br>• How much was the patient's Kt/V§§ value during the past month? (more than 1.2?)<br>• During the past month, were you informed about the results of your musculoskeletal tests?

*PCI= Percutaneous Coronary Intervention
**ER= Emergency Room
†DVT= Deep Vein Thrombosis
††EMS= Emergency Medical Service
‡IV= Intravenous
‡‡tPA= Tissue Plasminogen Activator
§ Behvarz = Low-cost primary healthcare workers
§§ Kt/V= It is a measure to assess dialysis adequacy.
